# Comparative efficacy of first-line therapeutic interventions for achalasia: a systematic review and network meta-analysis

**DOI:** 10.1007/s00464-020-07920-x

**Published:** 2020-08-27

**Authors:** Antonio Facciorusso, Siddharth Singh, Syed M. Abbas Fehmi, Vito Annese, John Lipham, Rena Yadlapati

**Affiliations:** 1 Gastroenterology Unit, Department of Medical Sciences, University of Foggia, 71100 Viale Pinto 1, Foggia, Italy; 2 Division of Gastroenterology, University of California San Diego, La Jolla, CA USA; 3 Division of Biomedical Informatics, University of California San Diego, La Jolla, CA USA; 4 Valiant Clinic & American Hospital, Dubai, United Arab Emirates; 5 Department of Surgery, University of Southern California, Los Angeles, CA USA

**Keywords:** POEM, Myotomy, Endoscopy, Pneumatic dilation

## Abstract

**Background:**

Several interventions with variable efficacy are available as first-line therapy for patients with achalasia. We assessed the comparative efficacy of different strategies for management of achalasia, through a network meta-analysis combining direct and indirect treatment comparisons.

**Methods:**

We identified six randomized controlled trials in adults with achalasia that compared the efficacy of pneumatic dilation (PD; *n* = 260), laparoscopic Heller myotomy (LHM; *n* = 309), and peroral endoscopic myotomy (POEM; *n* = 176). Primary efficacy outcome was 1-year treatment success (patient-reported improvement in symptoms based on validated scores); secondary efficacy outcomes were 2-year treatment success and physiologic improvement; safety outcomes were risk of gastroesophageal reflux disease (GERD), severe erosive esophagitis, and procedure-related serious adverse events. We performed pairwise and network meta-analysis for all treatments, and used GRADE criteria to appraise quality of evidence.

**Results:**

Low-quality evidence, based primarily on direct evidence, supports the use of POEM (RR [risk ratio], 1.29; 95% confidence intervals [CI], 0.99–1.69), and LHM (RR, 1.18 [0.96–1.44]) over PD for treatment success at 1 year; no significant difference was observed between LHM and POEM (RR 1.09 [0.86–1.39]). The incidence of severe esophagitis after POEM, LHM, and PD was 5.3%, 3.7%, and 1.5%, respectively. Procedure-related serious adverse event rate after POEM, LHM, and PD was 1.4%, 6.7%, and 4.2%, respectively.

**Conclusions:**

POEM and LHM have comparable efficacy, and may increase treatment success as compared to PD with low confidence in estimates. POEM may have lower rate of serious adverse events compared to LHM and PD, but higher rate of GERD.

**Electronic supplementary material:**

The online version of this article (10.1007/s00464-020-07920-x) contains supplementary material, which is available to authorized users.

Achalasia is a chronic disorder characterized by esophageal dysmotility and inadequate lower esophageal sphincter (LES) relaxation commonly manifesting with dysphagia and regurgitation. In the absence of a cure, the therapeutic goals in achalasia include symptom reduction and improved esophageal emptying [1, 2]. Endoscopic pneumatic dilation (PD) and laparoscopic Heller myotomy (LHM) often combined with an anti-reflux procedure have historically been the two definitive first-line therapies for achalasia, while peroral endoscopic myotomy (POEM) has emerged as an efficacious endoscopic therapy for achalasia over the past decade. Oral and endoscopic pharmacologic treatments are reserved as second-line options for patients that are not candidates for first-line therapy [3, 4].

PD is the most commonly performed treatment worldwide, and is minimally invasive with long-term success in 50–93% patients, although it usually requires several treatment sessions [5, 6]. On the other hand, LHM combined with an anti-reflux procedure is a more invasive treatment often requiring only one treatment session with success rates of 71% to –92% [7]. In the European Achalasia trial, the success rate of PD and LHM was shown to depend on achalasia subtype with type II achalasia having the highest success rate at 5 years (PD 96%; LHM 88%) and type III achalasia with the poorest success rate at 5 years (PD 48%; LHM 86%) [8]. The success rate of POEM in prospective cohorts has been greater than 90% and maintained across achalasia subtypes, thought to be related to the ability to perform a proximal extended myotomy in type III, or spastic achalasia. A consistent observation with POEM has been a higher risk of gastroesophageal reflux disease (GERD) compared to PD or LHM [9, 10].

In the last year, two landmark multicenter RCTs comparing POEM to PD and POEM to LHM have been published providing a framework for assessing comparative efficacy and safety of these interventions to inform optimal first-line intervention for treatment of patients with achalasia [11, 12]. Hence, we performed a pairwise and network meta-analysis combining direct (from RCTs directly comparing treatments of interest) and indirect evidence (from RCTs comparing treatments of interest with a common comparator), to compare the relative efficacy and safety of PD, LHM, and POEM for the management of achalasia. We used Grading of Recommendations Assessment, Development and Evaluation (GRADE) criteria for network meta-analysis to appraise quality of evidence [13].

## Methods

This systematic review is reported according to the Preferred Reporting Items for Systematic Reviews and Meta-Analyses (PRISMA) for network meta-analyses (PRISMA-NMA) statement and was conducted following a priori established protocol [14]. We also followed good research practices as outlined in the ISPOR (International Society for Pharmacoeconomics and Outcomes Research) report on interpreting indirect treatment comparisons and network meta-analysis for health-care decision making.

### Selection criteria

Studies included in this meta-analysis were RCTs with minimum follow-up of 1 year that met the following inclusion criteria: (a) Patients: adults (age > 18 years) with achalasia, treated with (b) Interventions and Comparators: PD, LHM, and POEM, and reported (c) Outcome: treatment success assessed at 1 year.

We excluded (a) observational or non-randomized studies, (b) RCTs of endoscopic botulinum toxin injection, as this is considered second-line therapy for patients who are not candidates for first-line therapy, (c) RCTs of oral therapies reserved for patients who are not candidates for first-line therapy (i.e., oral smooth muscle relaxants), and (c) trials with short duration of follow-up (< 1y) [15].

### Search strategy

The search strategy was conducted updating a prior systematic literature review performed as part of the recent American Society of Gastrointestinal Endoscopy (ASGE) guidelines [16] through December 2019. Briefly, in this guideline, combinations of subject headings and text words were used, including Esophageal Achalasia OR cardiospasm OR achalasia OR megaesophagus OR mega-esophagus OR megaoesophagus OR mega-oesophagus AND Botulinum Toxins OR botulin* OR botox OR myotomy OR Heller OR peroral OR per oral OR POEM OR LHM OR Dilatation/OR dilatation. Detailed search strategies can be viewed in the ASGE guidelines [16] and Supplementary Table 1.

**Table 1:** Characteristics of included randomized controlled trials comparing different interventions for management of achalasia

Study, year	Inclusion criteria	Location; Time period; Follow-up	Intervention, Number of patients	Control, Number of patients	Achalasia subtypes (I/II/III)	Definition of treatment success
Laparoscopic Heller myotomy vs. pneumatic dilation						
Boeckxstaens, 2011 [19]	Naïve, Eckardt > 3	Multicenter European; 2003–2008; 5 years	Laparoscopic Heller myotomy and Dor fundoplication; 106	Pneumatic dilation: at least two dilations, the first with 30 mm balloon and the second after 1 to 3 weeks with 35 mm balloon. In the case of symptoms recurrence, up to 2 additional series of dilations; 95	Intervention: 22 (20.7%)/61 (57.5%)/8(7.5%) Control: 22 (23.1%)/53 (55.7%)/10 (10.5%)	Eckardt score ≤ 3 at 1 year
Borges, 2014 [20]	Naive	Brazil; 2004–09; 3 years	Laparoscopic Heller myotomy and Dor fundoplication; 44	Pneumatic dilation with 30 mm balloon; 48	NR	According to Vantrappen and Hellemans: asymptomatic or dysphagia < 1/week
Hamdy, 2015 [22]	Naive	Egypt; 2005–10; 1 year	Laparoscopic Heller myotomy and Dor fundoplication; 25	Pneumatic dilation with 30 mm up to 40 mm balloon; 25	NR	Improvement of dysphagia (according to Demeester grading) at 1 year
Kostic, 2007 [21]	Naive	Sweden; 2000–05; 1 year	Laparoscopic Heller myotomy and Toupet fundoplication; 25	Pneumatic dilation: at least two dilations, the first with 30-mm balloon (35 mm in men) and the second after 1 with 35 mm balloon (40-mm in men); 26	NR	Absence of symptoms at 1 year
POEM vs. pneumatic dilation						
Ponds, 2019 [11]	Naïve, Eckardt > 3	Multicenter; 2012–2015; 2 years	POEM; 64	Pneumatic dilation: first dilation with 30 mm, eventual second dilation with 35 mm balloon; 66	Intervention: 10(16%)/42(65%)/12(19%) Control: 21(32%)/39(59%)/ 6(9%)	Eckardt score ≤ 3 at 2 years
POEM vs. Laparoscopic Heller myotomy						
Werner, 2019 [12]	Eckardt > 3	Multicenter; 2012–15; 2 years	POEM; 112	Laparoscopic Heller myotomy and Dor fundoplication; 109	Intervention: 15(13.4%)/ 85 (75.9%)/12(10.7%) Control: 21(19.2%)/78(71.5%)/1 (0.9%)	Eckardt score ≤ 3 at 2 years

*NR* not reported, *POEM* peroral endoscopic myotomy

An updated literature search of Pubmed and conference proceedings of Digestive Disease Week (DDW), United European Gastroenterology Week (UEG week), European Society of Gastrointestinal Endoscopy (ESGE) days was performed on December 20, 2019 to identify additional studies.

### Data abstraction and quality assessment

Data on study-, patient-, and treatment-related characteristics were abstracted onto a standardized form, by two authors independently (AF, RY). The risk of bias of individual studies was assessed in the context of the primary outcome, using the Cochrane Risk of Bias 2 assessment tool [17].

### Outcomes assessed

The primary efficacy outcome was treatment success at 1 year. Treatment success was defined based on decrease of Eckardt score (which measures symptom severity for dysphagia, regurgitation, retrosternal pain, and weight loss) [18] to ≤ 3 in 3 RCTs [11, 12, 19], absence of dysphagia in 2 RCTs (according to a specific questionnaire in the study by Borges et al. [20], or patient-reported improvement of symptoms in the study by Kostic et al. [21]), and according to DeMeester grading of dysphagia in a single RCT [22].

Secondary efficacy outcomes included treatment success at 2 years and physiologic outcomes (reduction in basal pressure of the lower esophageal sphincter [LES]; decrease in integrated relaxation pressure [IRP], post-treatment height of barium contrast on timed barium esophagram). Primary safety outcome was risk of post-treatment GERD at 1 year from the therapy; secondary safety outcomes were risk of severe erosive esophagitis (LA Grade C or D), and procedure-related serious adverse events.

### Statistical analysis

Direct meta-analysis was performed using the Mantel–Haenszel fixed-effects model (in the absence of conceptual heterogeneity and < 5 studies) to estimate pooled risk ratio (RR) and 95% confidence intervals (CI); with small number of studies, random effects models can be unstable [23].

We assessed statistical heterogeneity using I^2^ statistic, with values over 50% indicating substantial heterogeneity [24]. Due to the small number of trials, formal assessment of publication bias was not performed. Direct comparisons were performed using RevMan v5.3 (Cochrane Collaboration, Copenhagen, Denmark) (Review Manager (RevMan), ver. 5.3 ed: The Cochrane Collaboration, 2014). Next, we conducted network meta-analysis using a multivariate fixed-effects meta-regression as described elsewhere [25]. We used a frequentist approach based on a mixed-effects consistency model and provided a point estimate from the network along with 95% CI from the frequency distribution of the estimate.

The primary outcome (treatment success at 1 year) was analyzed using the network meta-analysis, while treatment success at 2 years was compared only through a direct meta-analysis. Pooled prevalence of procedure-related serious adverse events and of GERD outcomes was reported and pooled estimates, computed through the random effects model by DerSimonian and Laird test and expressed as mean and standard deviation, of physiological outcomes (reduction in basal pressure of LES, decrease in IRP, and post-treatment height of barium contrast on barium esophagogram) were calculated.

The quality of evidence derived from the pairwise and network meta-analysis was judged using the GRADE framework. In this approach, direct evidence from RCTs starts at high quality and can be rated down based on risk of bias, indirectness, imprecision, inconsistency (or heterogeneity) and/or publication bias, to levels of moderate, low, and very low quality (Supplementary Table 2). The rating of indirect estimates starts at the lowest rating of the two pairwise estimates that contribute as first-order loops to the indirect estimate but can be rated down further for imprecision or intransitivity (dissimilarity between studies in terms of clinical or methodological characteristics). If direct and indirect estimates were similar (i.e., coherent), then the higher of their rating was assigned to the network meta-analysis estimates.

**Table 2:** Direct, indirect, and combined comparison between the definitive treatments for management of achalasia concerning the treatment success at 1 year

Comparison	Direct comparison		Indirect comparison		Network meta-analysis	
	Risk ratio (95% CI)	Quality of evidence	Risk ratio (95% CI)	Quality of evidence	Risk ratio (95% CI)	Quality of evidence
Treatment success at 1 year						
LHM vs. PD	1.13 (0.90–1.40)	Low^a,b^	1.49 (0.90–2.43)	Very low	1.18 (0.96–1.44)	Low
POEM vs. PD	1.49 (1.01–2.21)	Low^a,b^	1.14 (0.79–1.64)	Very low	1.29 (0.99–1.69)	Low
POEM vs. LHM	1.01 (0.76–1.35)	Very low^a,c^	1.32 (0.84–2.08)	Very low	1.09 (0.86–1.39)	Very low

*PD* pneumatic dilation, *LHM* Laparoscopic Heller Myotomy; POEM Peroral Endoscopic Myotomy

^a^Evidence rated down for serious risk of bias

^b^Evidence rated down for serious imprecision (low event rate, such that optimal information size is not reached; 95% CI crossing unity for LHM vs. PD)

^c^Evidence rated down for very serious imprecision (very wide 95% CI)

## Results

### Studies

**Fig. 1:**
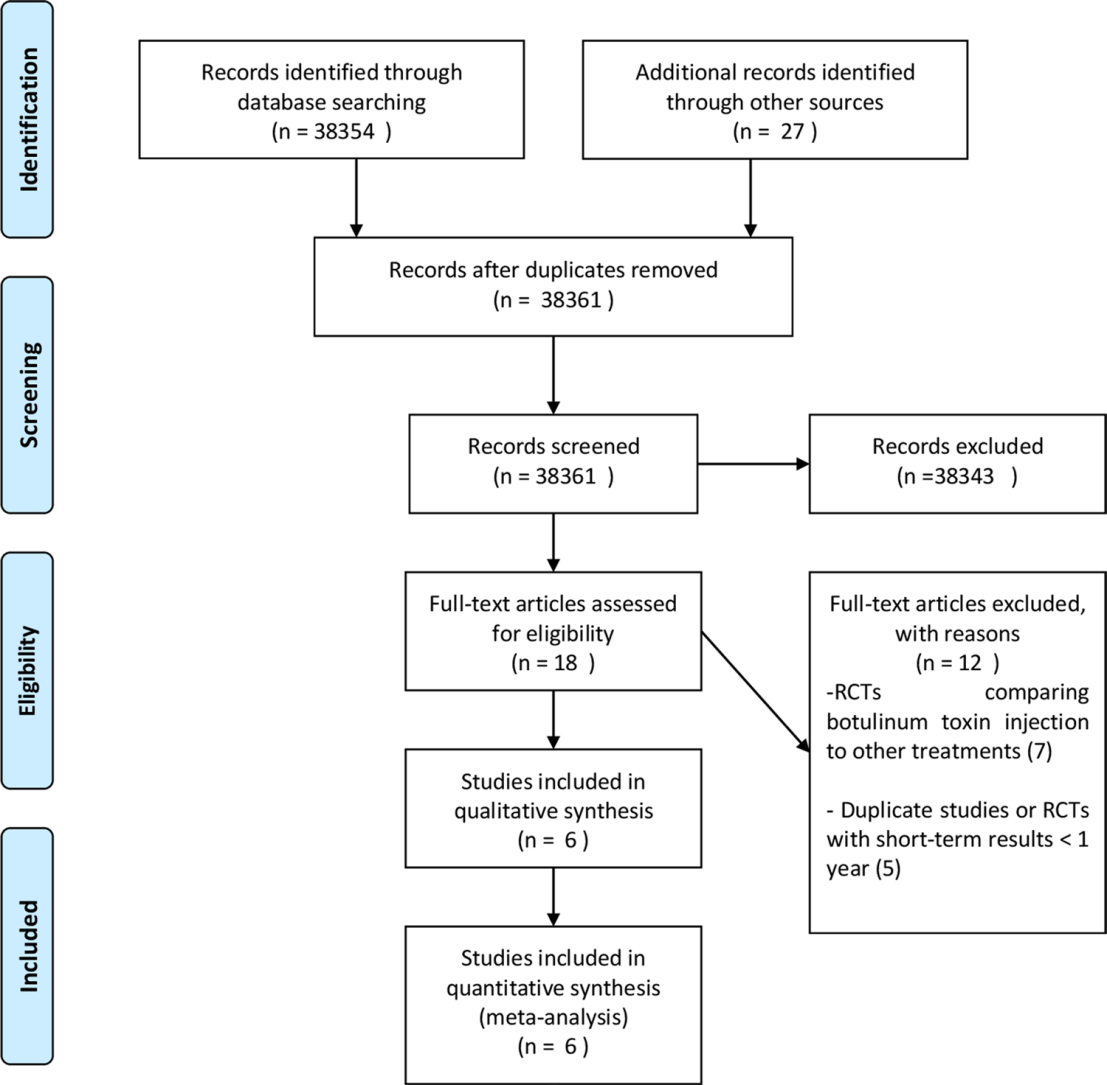
Flow chart of the included trials

From 38,354 unique studies identified using the search strategy, 6 RCTs met inclusion criteria and are included in the network meta-analysis to compare three different strategies for management of achalasia. Figure 1 shows the flow chart of study selection.

**Fig. 2:**
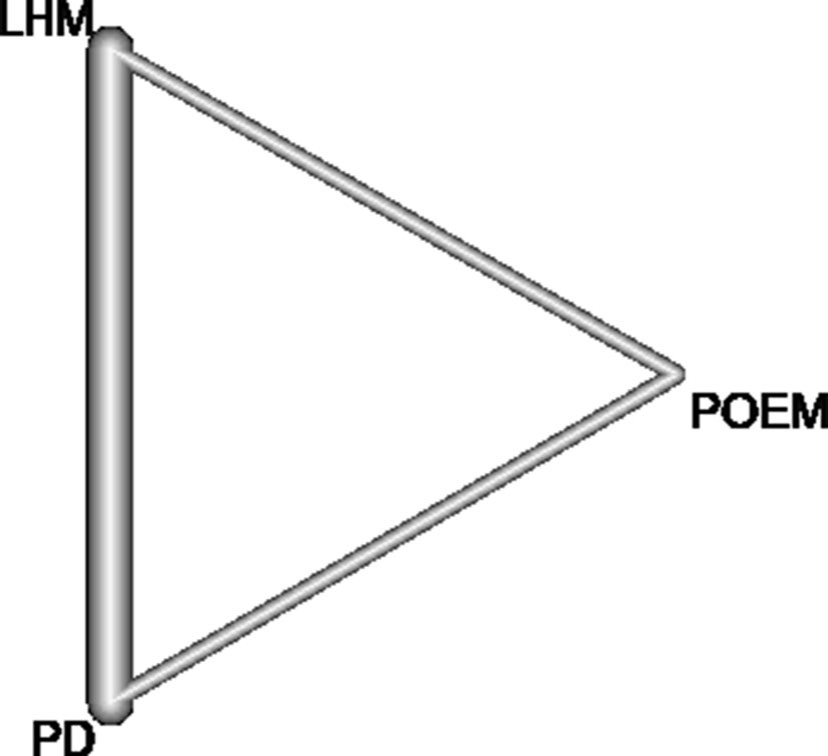
Network geometry of trials. Network of included studies with the available direct comparisons between interventions for management of achalasia. The size of the nodes and the thickness of the edges are weighted according to the number of studies evaluating each treatment and direct comparison, respectively. *PD* pneumatic dilation, *LHM* laparoscopic Heller myotomy, *POEM* peroral endoscopic myotomy

Table 1 summarizes the RCTs included in the network meta-analysis. Overall, these six trials included 745 patients. All six RCTs were two-arm controlled trials, in which four compared LHM with PD [19–22], one compared POEM to PD [11] and one compared POEM with LHM [12]. Overall, 309 patients were treated with LHM, 260 with PD, and 176 with POEM. The network of the included trials is reported in Fig. 2.

All RCTs enrolled treatment-naïve patients except for the RCT by Werner et al. [12], in which 35.7% patients were previously treated for achalasia (26.2% treated with PD, 6.7% with botulin toxin injection and 2.7% with combined PD and botulinum toxin). LHM was combined with Dor fundoplication in four studies [12, 19, 20, 22] and with Toupet fundoplication in one RCT [21].

The primary outcome (treatment success at 1 year) was reported in all studies, whereas only 4 RCTs reported treatment success at 2 years [12, 19, 20, 22].

Serious adverse events were not consistently defined or reported in the trials.

Demographical and clinical characteristics of trial patients are reported in the Supplementary Table 3. Baseline patient characteristics and prognostic factors (namely, age, gender, body mass index (BMI), baseline Eckardt score, baseline LES pressure) were comparably distributed in the intervention and comparator groups and across different trials. Achalasia subtypes according to manometry findings were reported in 3 RCTs [11, 12, 19] with 111 (20.1%) achalasia type I, 358 achalasia type II (64.8%), and 49 achalasia type III (8.8%).

**Table 3:** Clinical and objective evaluation of gastroesophageal reflux disease after treatment

Variable	Treatment	No. of Cohorts	No. of patients	Pooled rate (95% CI)
Daily reflux symptoms	PD	2	61	19% (9.2–28.8)
	LHM	2	128	13.5% (0–38.8)
	POEM	2	168	17.4% (0–39.9)
Endoscopic esophagitis	PD	2	161	14.7% (6.5–13.1)
	LHM	2	215	24.9% (16.4–33.3)
	POEM	2	176	45.4% (38.1–52.9)
Severe esophagitis (LA score C or D)	PD	2	161	1.5% (0–3.7)
	LHM	2	215	3.7% (0–8.1)
	POEM	2	176	5.3% (2–8.6)
Abnormal acid exposure	PD	2	108	20.4% (7.8–32.9)
	LHM	3	170	18.6% (2.5%-34.6%)
	POEM	1	70	30% (19.3–40.7)

Variable	Treatment	No. of Cohorts	No. of patients	Mean (95% CI)
Mean acid exposure time	PD	2	90	2.3% (1.5–3.1)
	LHM	2	127	4.3% (2.2–6.4)
	POEM	2	124	5.8% (5–6.6)

*CI* Confidence Interval, *LA score* Los Angeles score, *LHM* Laparoscopic Heller Myotomy, *PD* pneumatic dilation, *POEM* peroral endoscopic myotomy

Out of 745 patients enrolled in the included trials, 397 were male (53.2%) and baseline Eckardt score ranged from 6 to 9 while mean baseline LES pressure ranged from 23.9 to 39.8 mmHg.

Risk of bias assessment was performed in the context of the primary outcome. Due to lack of blinding of patients and physicians (outcome assessors) for a subjective outcome, studies were deemed to be at high risk of performance and detection bias (Supplementary Fig. 1).

### Comparative efficacy of first-line interventions

#### Treatment success at 1 year

**Fig. 3:**
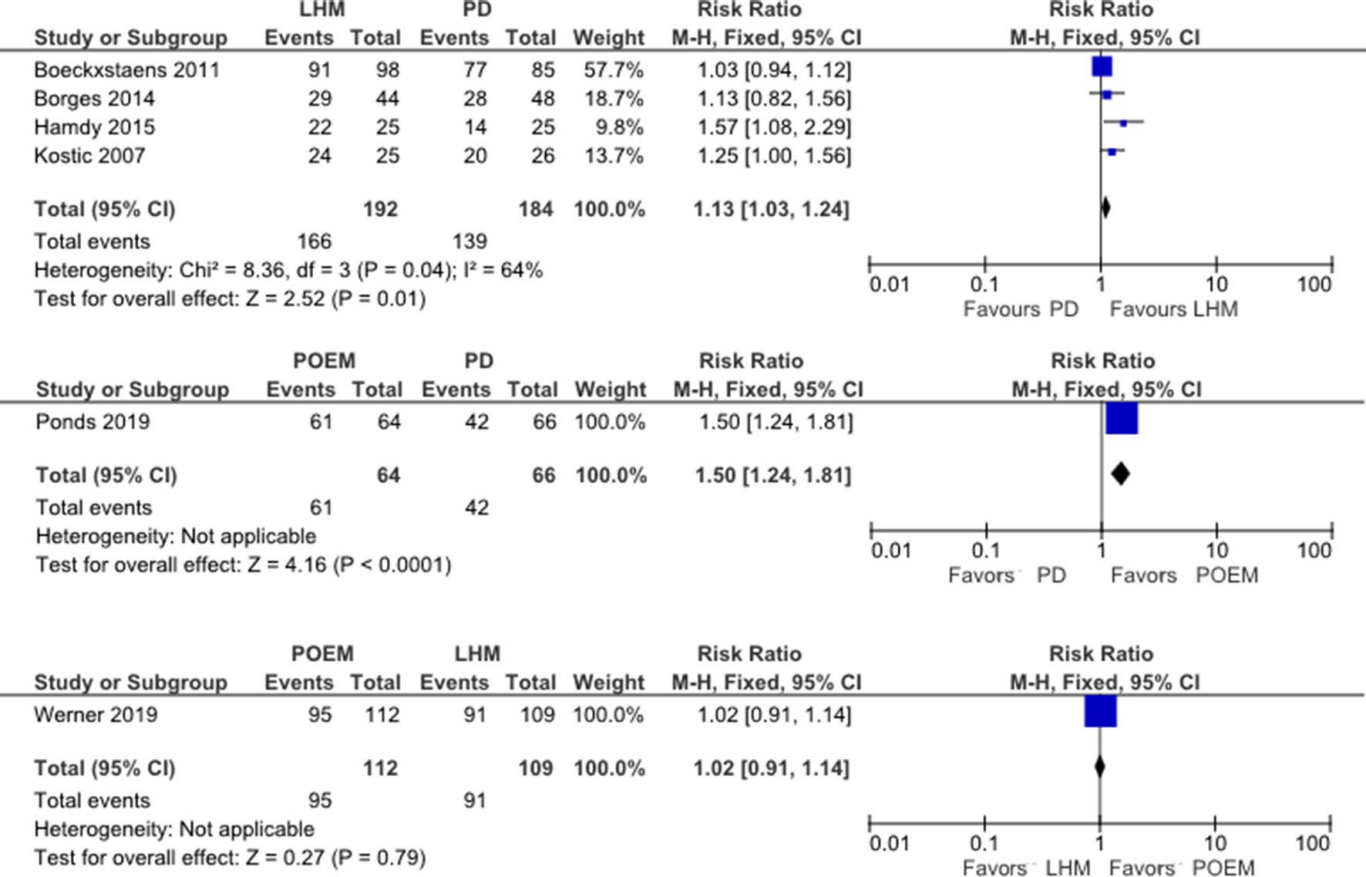
Direct meta-analysis comparing different treatment strategies for achalasia. Primary outcome was treatment success assessed at 1 year. *PD* pneumatic dilation, *LHM* laparoscopic Heller myotomy, *POEM* peroral endoscopic myotomy

Based on pairwise meta-analysis of 4 RCTs (376 patients) [19–22], LHM was more effective than PD (RR, 1.13; 95% CI 1.03–1.24; Fig. 3), albeit with considerable heterogeneity (*I*^2^ = 64%). Based on single RCTs, POEM was more effective than PD (RR, 1.50 [1.24–1.81]) [11] and similar to LHM [12] (RR, 1.02 [0.91–1.14]; Fig. 3). Evidence from direct estimates comparing LHM vs. PD and POEM vs. PD was rated down for serious risk of bias (lack of blinding) and serious imprecision (low event rate, with optimal information size threshold of 200 events not met).

On network meta-analysis, combining direct and indirect effect estimates, similar findings were observed. There was low confidence in estimates supporting higher efficacy of LHM vs. PD (RR, 1.18 [0.96–1.44]) and of POEM vs. PD (RR, 1.29 [0.99–1.69)]) (Table 2 and Supplementary Table 4). Quality of evidence, primarily based on direct evidence, was rated down due to serious risk of bias, and due to serious imprecision (lower limit of 95% CI crossing unity). No significant incoherence (differences in direct and indirect estimates) was observed in closed loops and no evidence of inconsistency was registered (Cochran’s Q test 2.37, p = 0.6684). No significant difference was observed in the efficacy of POEM vs. LHM, with very low confidence in estimates supporting the use of POEM and LHM due to very serious imprecision and serious risk of bias (Supplementary Table 4).

#### Secondary efficacy outcomes

##### Treatment success at 2 years

On direct meta-analysis, based on 2 RCTs (273 patients) [8, 20], there was no significant difference in treatment success at 2 years between LHM vs. PD (RR, 1.05 [0.94–1.16]; *I*^2^ = 0%) (Supplementary Fig. 2). POEM was more effective than PD at 2 years (RR, 1.76 [1.37–2.25]); Supplementary Fig. 2). The efficacy of LHM and POEM was comparable at 2 years (RR, 1.02 [0.90–1.15]).

##### Physiologic outcomes

Secondary physiologic outcomes assessed at 1 year are reported in supplementary table 5.

The decrease in LES basal pressure was of 18.5 mmHg ± 2.3 with PD (186 patients), 18.9 mmHg ± 4.0 after LHM (131 patients), and 17.1 mmHg ± 4.0 after POEM (64 patients). The decrease in integrated relaxation pressure (IRP) was of 10.3 mmHg ± 4.9 with PD (66 patients), 15.3 mmHg ± 5.6 after LHM (109 patients), and 16.3 mmHg ± 1 after POEM (176 patients).

At 1 year, no evidence of barium contrast retention was reported after PD and LHM, while the trial by Ponds et al. [11] reported 1.7 cm (interquartile range: 0–3.3) of barium contrast retention after POEM. At 2 years, 1.84 cm (0–8.8), 1.9 cm (0–6.8), and 2.3 cm (0–3.7) of barium contrast retention were registered after PD, LHM, and POEM, respectively (Supplementary Table 5).

### Comparative safety of first-line interventions

#### Risk of gastroesophageal reflux disease (GERD)

The clinical and objective evaluation of GERD are reported in Table 3.

The pooled rate of endoscopic evidence of esophagitis was 14.7% (95% CI: 6.5%–13.1%) after PD based on 2 RCTs [11, 19], 24.9% (16.4%–33.3%) after LHM based on 2 RCTs [12, 19], and 45.4% (38.1%–52.9%) after POEM based on 2 RCTs [11, 12]. The overall incidence of severe esophagitis after LHM, PD, and POEM was 3.7% (0%–8.1%), 1.5% (0%–3.7%), and 5.3% (2%–8.6%), respectively. Abnormal acid exposure was registered in 20.4% (7.8%–32.9%) of patients after PD, 18.6% (2.5%–34.6%) after LHM, and 30% (19.3%–40.7%) after POEM while mean acid exposure time was 2.3% (1.5%–3.1%), 4.3% (2.2%–6.4%), 5.8% (5%–6.6%) after PD, LHM, and POEM, respectively.

#### Risk of treatment-related serious adverse events

Supplementary Table 6 reports the incidence of serious adverse events observed in the included trials. Risk of serious procedure-related adverse events with LHM, PD, and POEM was 6.7% (95% CI: 1.4%–11.9%), 4.2% (1.8%–6.6%), and 1.4% (0%–3.2%), The most frequent serious complication after PD was perforation, which ranged from 1.5% to 8% of treated patients. In two RCTs [19, 22], 12% of patients treated with LHM experienced a severe mucosal injury, while the perforation rate for LHM was 4% in the study by Hamdy and colleagues [22] and 2.7% in the trial by Werner et al. [12]. Overall, perforation rate was 4.2% after PD and 1.2% with LHM, while none of the patients treated with POEM experienced this complication.

## Discussion

Treatment choice for achalasia represents a common challenge and a matter of debate in clinical practice. Though definitive endoscopic or surgical interventions have been studied, there has been limited synthesis of data on the comparative efficacy of these treatments, in particular since the development of POEM. Through a network meta-analysis, and using GRADE criteria to appraise quality of evidence, we made several key observations on the comparative efficacy and safety of these interventions. First, POEM and LHM may be more effective than PD, and comparable to each other, in decreasing achalasia-related symptoms at 1 year. Second, the risk of GERD was higher with POEM as compared to PD or LHM, but the risk of severe erosive esophagitis was low across all interventions. Overall risk of serious adverse events including perforation was lower with POEM as compared to PD or LHM.

Findings from this meta-analysis combined with consideration of relevant clinical factors can help guide clinicians and patients on treatment selection. Achalasia subtype is well established as critical prognostic factor. While we were unable to compare therapeutic efficacy across achalasia subtypes, prior experiences and studies suggest that efficacy in type III achalasia is greater following POEM with a proximal extended esophageal myotomy compared to LHM without extended myotomy or PD, due to the ability to treat not only the lower esophageal sphincter but also the spasticity in the distal esophageal smooth muscle. As such, the ASGE 2020 guideline on the management of achalasia, the American College of Gastroenterology 2020 guideline for Achalasia, as well as the Best Practice Advice from the American Gastroenterological Association Institute in 2017 recommend POEM as the preferred treatment for management of type III achalasia [16, 26].

Additional patient-specific and resource considerations are relevant to choice of therapy. A recent systematic review and meta-analysis by Oude Nijhuis and colleagues additionally identified older age and presence of sigmoid-shaped esophagus as poor predictors of treatment success [27]. Health-care utilization is additionally an important consideration. Compared to POEM, mean operative time, blood loss, and requirements for narcotics are generally greater with LHM and length of hospital stay is either similar or longer with LHM [28–30]. Overall, direct costs for both POEM and LHM do not significantly differ, though when considering quality adjusted life years, POEM appeared to be cost effective compared to LHM [31]. While PD utilizes fewer resources, patients should be aware that outcomes following PD are optimized with a sequential dilation protocol. In fact, in this network meta-analysis POEM remained more effective than PD at 2 years, though the observed efficacy of LHM vs PD was not apparent at 2 years. Discordancy in efficacy is likely driven by variation in PD protocols among studies, as depicted in Table 1, which challenges the ability to actually compare long-term efficacy outcomes. All things considered, the ultimate therapeutic decision should be patient centered based on shared decision-making models, and this is an area that requires further investigation and understanding.

An important strength of this network meta-analysis is the inclusion of RCTs alone and inclusion of new trials, in particular, the two landmark head-to-head RCTs comparing POEM to LHM and PD [11, 12]. Prior meta-analyses predated these RCTs, included both observational and randomized studies, and did not objectively appraise the overall quality of evidence using standardized GRADE methodology [7, 32].

Although the finding of increased rate of post-treatment GERD after POEM was expected, it is important to discuss the clinical implications. Ten to 20-year long-term follow-up after Heller myotomy reports high incidence of GERD and erosive complications including strictures, Barrett’s esophagus, and esophageal adenocarcinoma, which in turn contribute to the failure rate of myotomy at 10 years [33]. As such, Heller myotomy is often performed with an anti-reflux procedure. In POEM, the lack of a combined anti-reflux procedure to strengthen the integrity of the anti-reflux barrier [34] likely augments a gastroesophageal reflux physiology following POEM. However, the pooled estimate of severe erosive reflux disease following POEM in the short term is low. A previous meta-analysis found that approximately 30 patients should be treated with LHM over POEM to prevent 1 case of post-procedure severe esophagitis [34]. Further, initial experiences of endoscopic transoral incisionless fundoplication following POEM have been shown to reduce risks of esophagitis and esophageal acid exposure [35]. Nonetheless, long-term follow-up of consequences of GERD post POEM needs to be studied.

There are certain limitations, related to both the network meta-analysis as well as individual studies, which merit further discussion. The studies had a short duration of follow-up, and the primary outcome of this network meta-analysis was focused on short-term (1-year) success. Limited post-intervention follow-up prevents the ability to understand long-term comparative efficacy between interventions, which is critical for a chronic disease. There was also a paucity of direct head-to-head comparative trials, in particular comparing POEM to the other treatments. Further, performance and detection bias related to the non-blinded design of included trials introduced significant risk of bias. Performance and detection bias are not easily avoidable in RCTs testing new devices or techniques in surgery or endoscopy, given the nature of the intervention under study, and this represents a limitation in particular when considering subjective outcomes such as improvement of symptoms in patients with achalasia. Assessment of vital physiologic outcomes by blinded readers can overcome limitations related to blinding in these trials; however, these outcomes were infrequently and/or inconsistently reported in trials data which limit ability to compare post-treatment physiologic efficacy. Similarly, treatment-related adverse events were poorly reported and a thorough assessment of risk–benefit profile could not be performed. Finally, inherent to network meta-analyses is risk of misinterpretation due to conceptual heterogeneity, related to differences in participants, interventions, co-interventions/background treatment, and outcome assessment, which may limit comparability of trials; these cannot be adequately accounted for with study-level synthesis, and individual participant-level pooled analyses will be needed.

In conclusion, based on network meta-analysis, POEM and LHM may be comparable to each other, and both may be more effective than PD, in the definitive management of treatment-naïve patients with achalasia. While the risk of GERD is higher with POEM, overall rate of severe erosive esophagitis is low. Rate of treatment-related serious adverse events may be lower with POEM versus LHM or PD. Future prospective studies comparing long-term efficacy and safety of POEM, LHM, and PD, particularly across specific achalasia subtypes are warranted.

## Electronic supplementary material

Below is the link to the electronic supplementary material.

Supplementary file1 (DOCX 253 kb)
