# Supplementary material for: Comparative efficacy of first-line therapeutic interventions for achalasia: a systematic review and network meta-analysis
Source: Surg Endosc. 2020 Aug 27;35(8):4305–14. doi: 10.1007/s00464-020-07920-x (PMC8011535; doi:10.1007/s00464-020-07920-x)

**Supplementary Table 1. Embase search strategy used in the study.**

| Dilation  Query((((’esophagus achalasia’/exp) OR (’cardiospasm’:ab,ti OR ’achalasia’:ab,ti OR ’megaesophagus’:ab,ti OR ’mega  esophagus’:ab,ti OR ’megaoesophagus’:ab,ti OR ’mega oesophagus’:ab,ti)) AND ((’balloon dilatation’/exp) OR (dilation  OR dilatation:ab,ti)) AND [humans]/lim AND [english]/lim AND ([embase]/lim OR [embase classic]/lim)) NOT ’case  report’) NOT ((((’esophagus achalasia’/exp) OR (’cardiospasm’:ab,ti OR ’achalasia’:ab,ti OR ’megaesophagus’:ab,ti OR  ’mega esophagus’:ab,ti OR ’megaoesophagus’:ab,ti OR ’mega oesophagus’:ab,ti)) AND ((’balloon dilatation’/exp) OR (dilation  OR dilatation:ab,ti)) AND [humans]/lim AND [english]/lim AND ([embase]/lim OR [embase classic]/lim)) NOT ’case  report’) AND ([editorial]/lim OR [letter]/lim OR [note]/lim) |
| --- |
| Myotomy  Query((((’esophagus achalasia’/exp) OR (’cardiospasm’:ab,ti OR ’achalasia’:ab,ti OR ’megaesophagus’:ab,ti OR ’mega  esophagus’:ab,ti OR ’megaoesophagus’:ab,ti OR ’mega oesophagus’:ab,ti)) AND ((’myotomy’/exp) OR (myotomy OR heller  OR peroral OR (per AND oral) OR poem OR lhm)) AND [humans]/lim AND [english]/lim AND ([embase]/lim OR [embase  classic]/lim)) NOT ’case report’) NOT (((’esophagus achalasia’/exp) OR (’cardiospasm’:ab,ti OR ’achalasia’:ab,ti OR  ’megaesophagus’:ab,ti OR ’mega esophagus’:ab,ti OR ’megaoesophagus’:ab,ti OR ’mega oesophagus’:ab,ti)) AND ((’myotomy’/  exp) OR (myotomy OR heller OR peroral OR (per AND oral) OR poem OR lhm)) AND [humans]/lim AND [english]/  lim AND ([embase]/lim OR [embase classic]/lim)) NOT ’case report’ AND ([editorial]/lim OR [letter]/lim OR [note]/lim) |
| Reference: Khashab MA, Vela MF, Thosani N, Agrawal D, Buxbaum JL, Abbas Fehmi SM, Fishman DS, Gurudu SR, Jamil LH, Jue TL, Kannadath BS, Law JK, Lee JK, Naveed M, Qumseya BJ, Sawhney MS, Yang J, Wani S. ASGE guideline on the management of achalasia. Gastrointest Endosc. 2020;91(2):213–227. |

**Supplementary Table 2.** GRADE (Grading of Recommendations, Assessment, Development and Evaluation) categories of quality of evidence

| **GRADE Quality of Evidence** | **Meaning** | **Interpretation** |
| --- | --- | --- |
| **High** | We are very confident that the true effect lies close to that of the estimate of the effect. | Further research is VERY UNLIKELY to change our confidence in the estimate of effect |
| **Moderate** | We are moderately confident in the estimate of the effect; the true effect is likely to be close to the estimate of the effect, but there is a possibility that it is substantially different. | Further research is LIKELY to have an impact on our confidence in the estimate of effect and MAY change the estimate |
| **Low** | Our confidence in the estimate of the effect is limited; the true effect may be substantially different from the estimate of the effect. | Further research is VERY LIKELY to have an impact on our confidence in estimate of effect and is LIKELY to change the estimate |
| **Very Low** | We have very little confidence in the estimate of the effect; the true effect is likely to be substantially different from the estimate of the effect. | Any estimate of effect is very uncertain |

**Supplementary Table 3.** Demographical and clinical characteristics of patients enrolled in the included randomized controlled trials comparing different interventions for management of achalasia.

| **Study, Year** | **Age** | **Gender male** | **Body Mass Index** | **Baseline Eckardt Score** | **Baseline LES pressure (mmHg)** | **Patient Questionnaire** |
| --- | --- | --- | --- | --- | --- | --- |
| **Laparoscopic Heller Myotomy vs. Pneumatic Dilation** | | | | | | |
| Boeckxstaens, 2011 | Intervention: 45.5 (42.8-48.3)  Control: 46.4 (43.2-49.6) | Intervention: 57 (53.7%)  Control: 60 (63.1%) | Intervention: 25 (24-26)  Control: 24.6 (23.8-25.4) | Intervention: 7.4 (7-7.8)  Control: 7 (6.7-7.4) | Intervention: 31 (28-33)  Control: 33 (30-37) | Intervention: QLQ-OES24 39 (36-42)  Control: QLQ-OES24 36 (34-39) |
| Borges, 2014 | Intervention: 45.8 (18-74)  Control: 52.8 (26-75) | Intervention: 16 (36.4%)  Control: 26 (52%) | NR | NR | Intervention: 29.9 (9.8-56.2)  Control: 27.8 (5-62.2) | NR |
| Hamdy, 2015 | Intervention: 32  Control: 30.8 | Intervention: 8 (32%)  Control: 5 (20%) | NR | Severe dysphagia in 4 patients in each group | Intervention: 39.8  Control: 37.4 | NR |
| Kostic, 2007 | Intervention: 43±14  Control: 45±18 | Intervention: 11 (44%)  Control: 13 (50%) | Intervention: Weight 68 Kg±13  Control: Weight 68 Kg±12 | NR | Intervention: 17.9±7.2  Control: 23.9±14.8 | Intervention: GSRS: 2.5±1.4  Control: GSRS 3.4±1.6 |
| **POEM vs. Pneumatic Dilation** | | | | | | |
| Ponds, 2019 | Intervention: 47 (37-56)  Control: 50 (32-62) | Intervention: 33 (52%)  Control: 40 (61%) | Intervention: 23.2±3.7)  Control: 23.4±4.1 | Intervention: 8 (6-9)  Control: 7 (6-9) | Intervention: 31 (25-45)  Control: 32.8 (24-45) | Intervention: DSQoL 25 (22-27)  Control: DSQoL 24 (22-26) |
| **POEM vs. Laparoscopic Heller Myotomy** | | | | | | |
| Werner, 2019 | Intervention: 48.6 ± 14.9  Control: 48.6±14.6 | Intervention: 68 (60.7%)  Control: 60 (55%) | Intervention: 24.8±4.6  Control: 24.5±4.5 | Intervention: 6.8±2  Control: 6.7±2 | Intervention: 26.8±11.4    Control: 26±10.9 | Intervention: GQoL 89.2±23.1  Control: GQoL 90.4±18.1 |

[Abbreviations: LES-Lower Esophageal Sphincter; NR-Not reported, POEM- Peroral Endoscopic Myotomy]

**Supplementary Table 4.** GRADE Summary of Findings reporting the comparative efficacy of different treatment for achalasia. Quality of the evidence was rated based on GRADE methodology. RCTs of direct comparison were rated down for presence of any of the following factors – risk of bias in literature, inconsistency, indirectness, imprecision and publication bias.

|  | **1-year treatment success** | |
| --- | --- | --- |
|  | Relative risk (95% CI) | Quality of Evidence |
| **All treatments vs. pneumatic dilation** | | |
| LHM | 1.18 (0.96-1.44) | Low |
| POEM | 1.29 (0.99-1.69) | Low |
| **vs. Laparoscopic Heller myotomy** | | |
| POEM | 1.09 (0.86-1.39) | Very Low |
| Abbreviations: LHM-Laparoscopic Heller Myotomy; POEM-Per Oral Endoscopic Myotomy | | |

**Supplementary Table 5. Physiological outcomes reported at 1 year**

|  |  | **Baseline** | | | **Post-Treatment** | | | **Mean difference** | | |
| --- | --- | --- | --- | --- | --- | --- | --- | --- | --- | --- |
| **Variable** | **Treatment** | **No. of Cohorts** | **No. of patients** | **Mean mmHg (standard deviation)** | **No. of Cohorts** | **No. of patients** | **Mean mmHg (standard deviation)** | **No. of Cohorts** | **No. of patients** | **Mean mmHg (standard deviation)** |
| **Baseline LES basal pressure** | **PD** | 5 | 260 | 30.9±4.6 | 3 | 186 | 11.4±2.3 | 3 | 186 | - 18.5 ±2.3 |
|  | **LHM** | 5 | 309 | 28.9±7.1 | 2 | 131 | 10±3.1 | 2 | 131 | -18.9±4 |
|  | **POEM** | 2 | 176 | 28.9±2.1 | 1 | 64 | 11.8±6.7 | 1 | 64 | -17.1 ±4.5 |
| **IRP** | **PD** | 2 | 91 | 21.3±7.2 | 1 | 66 | 11±2.3 | 1 | 66 | -10.3±4.9 |
|  | **LHM** | 2 | 134 | 26.8±6.4 | 1 | 109 | 11.5±0.8 | 1 | 109 | -15.3±5.6 |
|  | **POEM** | 2 | 176 | 26.6±4 | 2 | 176 | 10.3±3 | 2 | 176 | -16.3±1 |
|  |  | **Baseline** | | | **1 year after treatment** | | | **2 years after treatment** | | |
|  |  | **No. of Cohorts** | **No. of patients** | **Median cm (IQR)** | **No. of Cohorts** | **No. of patients** | **Median cm (IQR)** | **No. of Cohorts** | **No. of patients** | **Median cm (IQR)** |
| **Height of barium contrast** | **PD** | 2 | 161 | 9.35 (3-10.1) | 2 | 151 | 0 (0 to 1.5) | 2 | 119 | 1.84 (0-8.8) |
|  | **LHM** | 1 | 106 | 12 (8.1-18) | 1 | 98 | 0 (0 to 0.65) | 1 | 97 | 1.9 (0-6.8) |
|  | **POEM** | 1 | 64 | 7.2 (4.5-9.2) | 1 | 64 | 1.7 (0-3.3) | 1 | 58 | 2.3 (0-3.7) |
| Abbreviations: IQR, Interquartile Range; IRP, Integrated Relaxation Pressure; LES, Lower Esophageal Sfincter; LHM, Laparoscopic Heller Myotomy; NR, Not Reported; PD, Pneumatic Dilation; POEM, Per-Oral Endoscopic Myotomy | | | | | | | | | | |

**Supplementary Table 6.** Serious adverse events reported in the randomized controlled trials comparing different interventions for management of achalasia.

| **Study** | **Adverse Events in the Intervention Group** | **Adverse Events in the Control Group** |
| --- | --- | --- |
| **Laparoscopic Heller Myotomy vs. Pneumatic Dilation** | | |
| Boeckxstaens, 2011 | Mucosal tear 12%  Conversion to open surgery: 0.9% | Perforation: 4% |
| Borges, 2014 | None | Perforation: 4% |
| Hamdy, 2015 | Mucosal injury: 12%  Perforation: 4% | Perforation: 8% |
| Kostic, 2007 | None | Perforation: 7.7% |
| **Peroral Endoscopic Myotomy vs. Pneumatic Dilation** | | |
| Ponds, 2019 | None | Perforation: 1.5%  Severe Pain: 1.5% |
| **Peroral Endoscopic Myotomy vs. Laparoscopic Heller Myotomy** | | |
| Werner, 2019 | Mucosal Leakage: 0.9%  Aspiration: 0.9%  Pneumothorax: 0.9% | Perforation: 2.7%  Severe Pain: 1.8%  Pneumothorax: 1.8%  Aspiration: 0.9%  Hiatal Stenosis: 0.9% |

**Supplementary Figure 1.** Risk of bias summary of the included randomized-controlled trials


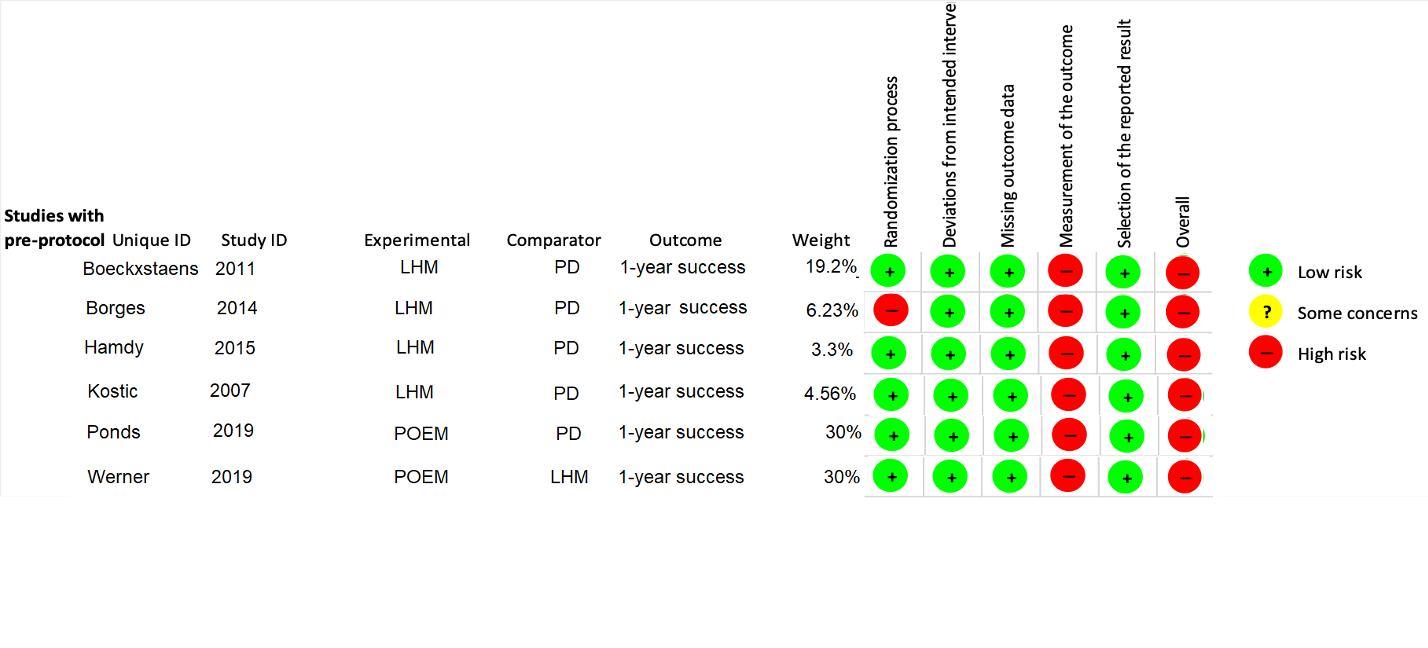


**Supplementary Figure 2.** Direct meta-analysis for 2-year treatment success rate


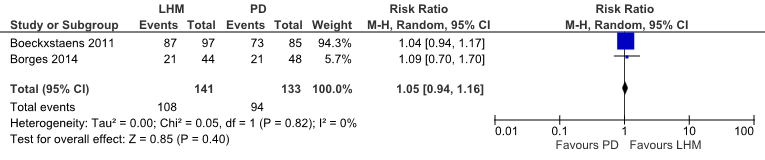


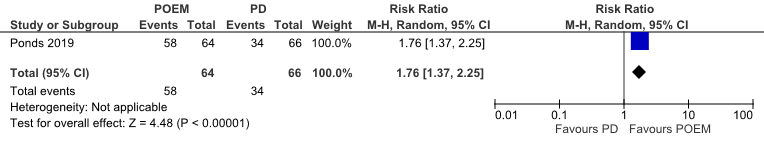

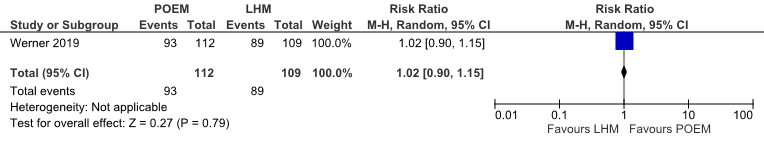

Supplement: Supplementary file 1 — Supplementary file1 (DOCX 253 kb) [file 464_2020_7920_MOESM1_ESM.docx]
